# Hypoxia Can Induce Migration of Glioblastoma Cells Through a Methylation-Dependent Control of *ODZ1* Gene Expression

**DOI:** 10.3389/fonc.2019.01036

**Published:** 2019-10-10

**Authors:** Carlos Velásquez, Sheila Mansouri, Olga Gutiérrez, Yasin Mamatjan, Pilar Mollinedo, Shirin Karimi, Olivia Singh, Nuria Terán, Juan Martino, Gelareh Zadeh, José L. Fernández-Luna

**Affiliations:** ^1^Department of Neurological Surgery and Spine Unit, Hospital Universitario Marqués de Valdecilla and Instituto de Investigación Marqués de Valdecilla (IDIVAL), Santander, Spain; ^2^MacFeeters-Hamilton Centre for Neuro-Oncology Research, Princess Margaret Cancer Centre, Toronto, ON, Canada; ^3^Genetics Unit, Hospital Universitario Marqués de Valdecilla and Instituto de Investigación Marqués de Valdecilla (IDIVAL), Santander, Spain; ^4^Department of Pathology, Hospital Universitario Marqués de Valdecilla and Instituto de Investigación Marqués de Valdecilla (IDIVAL), Santander, Spain; ^5^Division of Neurosurgery, Toronto Western Hospital/University Health Network, University of Toronto, Toronto, ON, Canada

**Keywords:** glioblastoma, methylation, ODZ1, migration, hypoxia, teneurin

## Abstract

The transmembrane protein ODZ1 has been associated with the invasive capacity of glioblastoma (GBM) cells through upregulation of RhoA/ROCK signaling, but the mechanisms triggering the ODZ1 pathway remain elusive. In addition, it is widely accepted that hypoxia is one of the main biological hallmarks of the GBM microenvironment and it is associated with treatment resistance and poor prognosis. Here we show that hypoxic tumor regions express higher levels of ODZ1 and that hypoxia induces ODZ1 expression in GBM cells by regulating the methylation status of the ODZ1 promoter. Hypoxia-induced upregulation of ODZ1 correlates with higher migration capacity of GBM cells that is drastically reduced by knocking down ODZ1. *In vitro* methylation of the promoter decreases its transactivation activity and we found a functionally active CpG site at the 3'end of the promoter. This site is hypermethylated in somatic neural cells and mainly hypomethylated in GBM cells. Mutagenesis of this CpG site reduces the promoter activity in response to hypoxia. Overall, we identify hypoxia as the first extracellular activator of ODZ1 expression and describe that hypoxia controls the levels of this migration-inducer, at least in part, by regulating the methylation status of the ODZ1 gene promoter.

## Introduction

Glioblastoma (GBM) is the most common primary malignant brain tumor and carries a dismal prognosis despite the current standard of care (1, 2). The invasive properties of GBM hamper the effect of treatment and lead to tumor recurrence. As such, the molecular pathways that regulate glioma cell invasion play a major role in the pathogenicity of GBM (3). Infiltrative GBM stem-like cells have been described in the peritumoral parenchyma (4) and their role in developing resistance to chemo- and radiotherapy is widely accepted (5, 6). Another hallmark of GBM is hypoxia, that has been associated with worse prognosis and treatment resistance and its role in GBM cell invasion has been extensively described (7–9).

Hypoxia triggers a complex tumor cell response that enables migration and invasion of the surrounding parenchyma through activation of multiple molecular pathways, as PI3K/Akt, Wnt/ß-catenin, Hedegehog, TGFß, and Tyrosine kinase receptors, among others (10–12). Hypoxia alters expression of these genes through biding of HIF to promoters of genes containing HREs (7). Nevertheless, to the best of our knowledge, the role of hypoxia in regulating migration of GBM cells through gene promoter methylation has not been completely addressed, despite that hypoxia is known to change the DNA methylation status (13).

ODZ1 (Teneurin-1, TNM1, TENM1), a philogenetically conserved type II transmembrane protein, has been associated to the migratory capacity of GBM cells by upregulating the RhoA-ROCK pathway that results in cytoskeletal remodeling, cell migration and invasion of peritumoral parenchyma (14). However, the extracellular triggers capable to upregulate ODZ1 have not yet been described.

In the present study, we found that hypoxia is able to induce the expression of ODZ1 in GBM cells and that the methylation status of the *ODZ1* promoter plays a major role in regulating the levels of this cell migration inducer.

## Materials and Methods

### Patients

A total of 17 patients with confirmed primary IDHwt GBM were included in the analysis. All of these patients in this cohort met all the following inclusion criteria: (1) age>18 years and (2) Histological confirmation of IDH wild-type GBM. Pimonidazole hydrochloride (PIMO) (Hpoxyprobe-1; Natural Pharmacia International Inc., Burlington, MA), an exogenous hypoxia marker with an IND (Investigational New Drug) status for use in the clinical evaluation of hypoxia, was administered to all patients 16–20 h prior surgical resection. Tumor specimens were obtained at the time of surgery and processed for further analysis. Approval of Research Ethics Board from University Health Network (Toronto, Canada) or Hospital Universitario Marques de Valdecilla (Santander, Spain) was obtained for each patient included in the study in accordance with The Code of Ethics of the World Medical Association (Declaration of Helsinki) for experiments involving humans.

### Immunohistochemical Staining and Analysis

We reviewed the H&E slides and select the best blocks with highest tumor cellularity aiming at 70% tumor cellularity. Immunohistochemical staining was performed using an in-house anti-icODZ1 antibody against the N-terminal region of ODZ1 (14) and Hypoxyprobe, a peroxidase-based immunostaining kit containing an anti-PIMO monoclonal antibody (NPI Inc., Burlington, MA). Both were used to assess ODZ1 cytoplasmatic and nuclear expression and stable cytoplasmic and nuclear PIMO protein adducts, respectively. Consecutive formalin-fixed paraffin-embedded tumor sections were stained and reviewed with each antibody. PIMO uptake and ODZ1 nuclear and cytoplasmic expression were quantified using a pixel-based image analysis software (Aperio ImageScope). PIMO negative and positive regions were delineated and annotated. Then, ODZ1 expression was determined in each annotated PIMO positive and PIMO negative region. PIMO and ODZ1 positivity cut-offs were defined as 15 and 5% in the tumoral areas, respectively.

### Promoter Methylation Status and Bioinformatics Analysis

In order to assess the methylation status of *ODZ1* promoter within hypoxic tumor cells, we dissociated fresh GBM specimens from 10 patients administered with PIMO 16–20 h prior to surgery. Cells were labeled with a FITC-conjugated PIMO-specific antibody, followed by FACS. DNA was isolated from FACS-sorted PIMO positive and negative cells. Methylation profiling was performed using the Illumina Infinium HumanMethylationEPIC Array (Illumina Inc., San Diego, CA). Raw data files (^*^.idat) were imported preprocessed and normalized with the ssNoob method using the minfi package (version 1.28.3) (15) from the Bioconductor package (version 3.8) (16) together with appropriate quality control (detection *P* < 0.05) and analysis procedure. Methylation values (beta-values) of all CpG sites were obtained which range from 0 for unmethylated to 1 for fully methylated. In addition, we analyzed the *ODZ1* methylation status in 155 GBM samples obtained from The Cancer Genome Atlas (TCGA) following a protocol previously described (17). Briefly, the TCGA 450k methylation data set (level 3) and clinical information were downloaded from the National Cancer Institute Genomic Data Commons. Four CpGs sites located within the *ODZ1* gene were included in the analysis: cg08750326, cg24761295, cg01792733, and cg19331065. Overall methylation in GBM cells cultured under hypoxia was assessed by using the colorimetric MethylFlash Global DNA Methylation ELISA kit (Epigentek, Farmingdale, NY) following the manufacturer protocol.

### Primary Cells Cultures

Primary GBM cells used in this study were previously established in our laboratory (14, 18). Three different primary cell lines established from tumor specimens of patients with GBM were used (G196, G52, and G63). Two of them did not express IDH1 mutant protein, overexpressed EGFR and were GFAP (+) while the other expressed mutant p53 protein. The tumor cells were maintained as neurospheres in serum-free DMEM/F12 medium (Invitrogen, Carlsbad, CA) and plated at a density of 3 × 10^6^ live cells/60-mm plate. Neurospheres were dissociated every 4–5 days to facilitate cell growth. Cells were used between passages 10 and 20. GBM cells were incubated under hypoxia (1% O_2_), in a Hypoxia Incubator Chamber (StemCell Technologies, Vancouver, BC, Canada), or normoxia (21% O_2_) and harvested after 24 or 48 h for further analysis. When indicated cells were treated with Hypoxiprobe-1 and immunolabeled with anti-PIMO to confirm an effective cellular response to hypoxia.

### Expression Analyses

The expression of individual genes was evaluated by quantitative RT-PCR on total cellular RNA as previously described (14). cDNA was generated and amplified using the following primers: β*-Actin* (5′GCGGGAAATCGTGCGTGACATT3′ and 5′GATGGAGTTGAAGGTAGTTTCGTG3′) and *ODZ1* (5′ACTCAAGAGATGGAATTCTGTG3′ and 5′CTTAGTGCATGGTCAGGTG 3′). qRT-PCR was performed in a 7000-sequence detection system (Life Technologies, Carlsbad, CA).

### Enzymatic Methylation of the *ODZ1* Promoter

A CpG methyl-transferase (M.SssI) was used to methylate all CpG sites in the *ODZ1* promoter according to manufacturer instructions (New England Biolabs, Ipswich, MA). Methylated promoter was cloned into KpnI and HindIII sites of an unmethylated pGL2-luciferase reporter plasmid (Promega, Madison, WI). DNA was isolated from transduced bacterial colonies and those maintaining the methylated *ODZ1* promoter were identified by digestion with the methylation-sensitive HpaII restriction enzyme (New England Biolabs) followed by PCR-amplification with primers flanking the CCGG site of interest (cg24761295) (5′TGCTGCAACCTCCAGCTTAAT3′ and 5′TGTGAGGAAATGCATCTGGCA3′). As a control, we amplified a fragment of the *ODZ1* promoter without HpaII sites with primers (5′TGCAACAGTGGACTGAAATGG3′ and 5′TCTTAGGGCCAGTAGAGGCAT3′).

### Transfections, Gene Reporter Assays, and Gene Silencing

GBM cells were cotransfected with 2 μg wild type and mutant promoter or methylated and unmethylated promoter cloned into pGL2-luciferase reporter, and 0.2 μg pRSV-ß-gal by using nucleofection. Transfected cells were cultured under hypoxic or normoxic conditions for 48 h and cell extracts were prepared and analyzed for the relative luciferase activity by a dual-light reporter gene assays (Applied Biosystems, Foster City, CA). Results were normalized for transfection efficiency with values obtained with pRSV-β-gal. Site-directed mutagenesis of cg24761295 in the OIDZ1 promoter was performed by using the QuickChange site-directed mutagenesis kit (Agilent Technologies, Santa Cruz, CA) with the following primers (5′CAGAGTGTTATTATTGCCTCAGGCTAGCTTCATGTCATCTAG3′ and 5′CTAGATGACATGAAGCTAGCCTGAGGCAATAATAACACTCTG3′). The modified promoter was sequenced to verify the mutation. When indicated, GBM cells were transfected with *ODZ1*-specific shRNAs (Thermo Fisher Scientific, Waltham, MA) by using nucleofection.

### Migration Assay

The migration capacity of GBM cells was analyzed by using a modified Boyden chamber assay in 24-well plates (Transwell, Corning Incorporated, NY). Cells were placed in the upper compartment and following 24 or 48 h of incubation under hypoxic and normoxic conditions, migratory cells in the lower face of the membrane were fixed and stained.

### Statistical Analysis

All statistics were calculated with the SPSS statistical package (version 13.0). Data are presented as mean ± SD of three independent experiments. Differences between groups were tested for statistical significance using the unpaired 2-tailed Student's *t*-test or Chi Square accordingly. The significance level was set at *p* < 0.05.

## Results

### Hypoxia Upregulates ODZ1 in GBM Tumor Specimens

**Figure 1:**
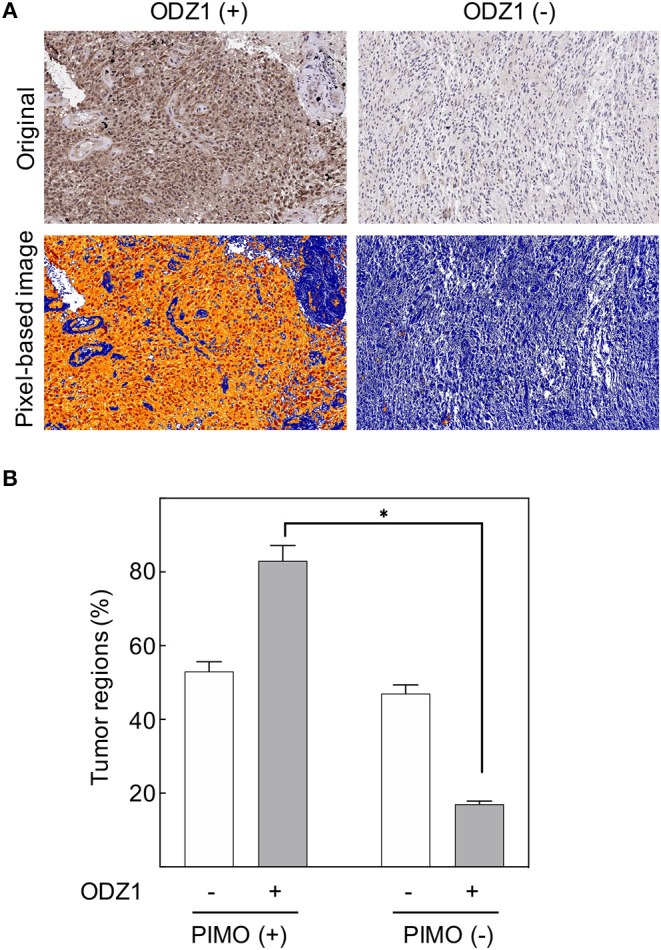
*In vivo* ODZ1 expression and its association with the hypoxic microenviroment in GBM. **(A)** GBM tissue specimens were immunostained with anti-ODZ1 antibody. Original and pixel-based image analysis of ODZ1 nuclear and cytoplasmic expression in positive and negative representative cases, are presented. **(B)** PIMO and ODZ1 immunostaining was determined in consecutive histological sections in 54 tumor regions. (Chi-Square, *p < 0.05).

Tumor cell migration is known to be triggered by microenvironmental stress including hypoxia, which is one of the hallmarks of GBM. Recently, we described ODZ1 as a novel inducer of migration/invasion in GBM (14) but the external stimuli that promote the expression of *ODZ1* are not known. To determine whether *ODZ1* expression was linked to hypoxia in GBM, we first examined the cellular uptake of the exogenous hypoxia marker pimonidazole (PIMO) administered to the patients prior to surgery to delineate the severely hypoxic tumor regions. We then analyzed the expression of ODZ1 protein in hypoxic and normoxic regions (Figure 1A). ODZ1 nuclear and cytoplasmic expression increased in the severe hypoxic regions when compared with the normoxic tumor regions in GBM. Up to 80% of regions that stained positive for the hypoxia marker PIMO were ODZ1-positive, whereas among the PIMO-negative regions <20% were ODZ1-positive (*p* < 0.05) (Figure 1B).

### Hypoxia Upregulates *ODZ1* Transcription and Promotes Migration of GBM Cells

**Figure 2:**
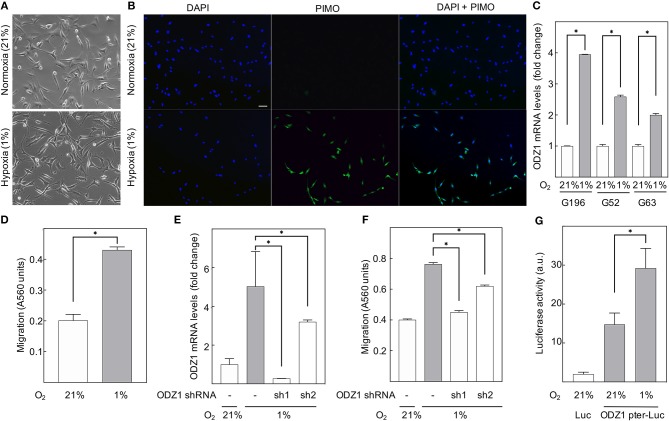
Hypoxia upregulates ODZ1 and promotes ODZ1-dependent migration of GBM cells. **(A)** Primary GBM cells cultured under hypoxia for 48 h maintain their phenotype with a viability higher than 80%. **(B)** Cells cultured in 1% O_2_ for 48 h stained positive for the hypoxia marker PIMO as confirmed by immunofluorescence with anti-PIMO antibody (more than 95% of PIMO positive cells). **(C)** ODZ1 mRNA levels in GBM cells under hypoxia (1% O_2_) and normoxia (21% O_2_) for 24 h were analyzed by qPCR in three different GBM primary cell lines (G196, G52, and G63). **(D)** A modified Boyden Chamber assay was used to assess cell migration under hypoxia and normoxia for 48 h. **(E)** Downregulation of hypoxia-induced ODZ1 mRNA levels in G196 cells transfected with two ODZ1-specific shRNAs and cultured under hypoxia for 48 h. **(F)** Cell migration capacity under hypoxia (48 h) in the presence of ODZ1 shRNAs. **(G)** Cells transfected with ODZ1 promoter cloned into a luciferase reporter plasmid were analyzed for luciferase activity under hypoxia (48 h). All histograms show the mean ± SD of three independent experiments. Student *t*-test *p < 0.05. Scale bar: 10 μm.

To determine whether hypoxia leads to increased expression of *ODZ1*, we cultured primary GBM cells under hypoxic conditions (1% O_2_) and found that the majority of the cells remained viable (over 85% by Trypan blue assay). We treated cells with PIMO before harvesting and performed immunofluorescence analysis to confirm that cells cultured in hypoxic conditions were able to uptake PIMO from culture media, while those grown in normoxia did not (Figures 2A,B). Consistent with our previous findings, analysis of *ODZ1* expression in hypoxia indicated a 4-fold increase in *ODZ1* mRNA expression (*p* < 0.05) (Figure 2C). Moreover, as shown in Figure 2D, hypoxia promoted migration of GBM cells (more than 2-fold increase, *p* < 0.05). In order to study whether ODZ1 contributed to hypoxia-induced migration, we knocked down ODZ1 by using two specific shRNAs and showed that efficient downregulation of *ODZ1* mRNA levels correlated with reduced migration of GBM cells under hypoxia (*p* < 0.05) (Figures 2E,F). Similar results were obtained with another GBM cell line (Supplementary Figure 1). Then, we cloned the *ODZ1* promoter into a luciferase reporter-containing plasmid. GBM cells transfected with this construct increased the level of luciferase activity when cultured in low oxygen (1-fold increase, *p* < 0.05) (Figure 2G) further demonstrating that hypoxia was relevant for the expression of *ODZ1* and that the promoter cloned contained hypoxia-responsive elements.

### *ODZ1* Promoter Methylation Status Is Modified by Hypoxia

**Figure 3:**
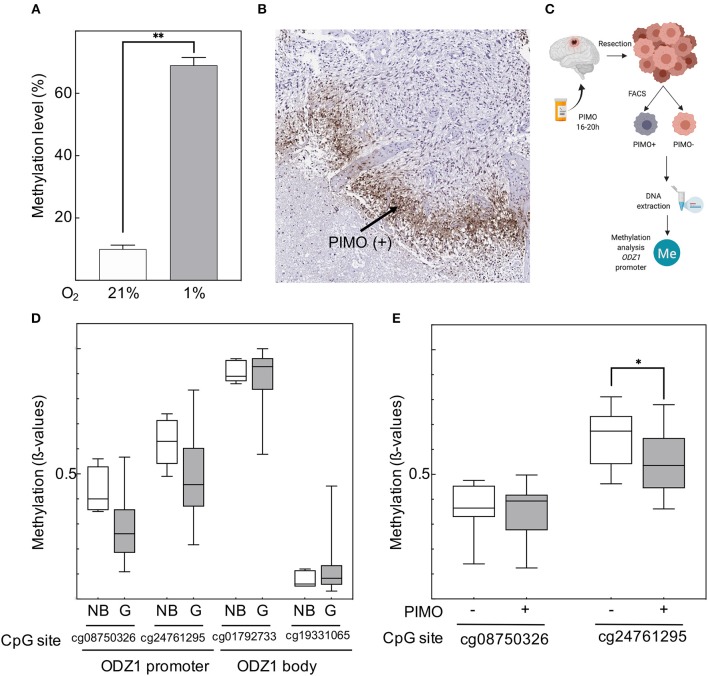
Hypoxia-dependent regulation of ODZ1 promoter methylation. **(A)** Overall methylation level of DNA from GBM cells cultured under hypoxic and normoxic conditions (48 h). **(B)** Immunohistochemistry of GBM tumor tissue with anti-PIMO antibody showing the presence of PIMO positive cells. **(C)** Schematic diagram of the experimental design to analyse the methylation status of CpG sites within the ODZ1 promoter in tumor tissue from GBM patients (Created with BioRender.com). **(D)** Methylation status of CpG sites in the ODZ1 gene in GBM tissue (*n* = 155) and normal brain (*n* = 5). Data obtained from TCGA. **(E)** Methylation of CpG sites located at the ODZ1 promoter in PIMO negative and PIMO positive cells in tumor specimens from 10 patients following the experimental design described in **(C)**. Histograms show the mean ± SD of three independent experiments. Boxes show the median with whiskers extended to minimum and maximum values. Student *t*-test *p < 0.05, **p < 0.01.

Hypoxia resulted in overall DNA methylation in GBM tumor cells (Figure 3A). We isolated PIMO positive and PIMO negative tumor cells from surgical specimens of 10 patients with GBM by using a cell sorter (Figures 3B,C) and analyzed the methylation status of the *ODZ1* promoter. We focused on a fragment of 1.4 kb upstream of the transcription start site that contained no canonical CpG island but 18 CpG sites. Analysis of 155 GBM cases from TCGA showed that two of these CpG sites (cg08750326 and cg24761295) were hypomethylated in GBM compared to normal brain tissue, while two other CpG sites (cg01792733 and cg19331065), located in the body of the gene, far from the transcription start site, had similar methylation status both in normal brain and GBM (Figure 3D). Kaplan Meier survival analysis based on the methylation status showed a tendential, but not statistically significant, correlation of cg24761295 site hypomethylation and poor survival (Supplementary Figure 2). The methylation status of CpG sites in the *ODZ1* promoter (cg08750326 and cg24761295) was analyzed in sorted PIMO positive and negative cells and found that only the cg24761295 was differentially methylated being hypomethylated in PIMO positive cells (Figure 3E), which is consistent with a higher expression of ODZ1 under hypoxia.

### Promoter Methylation or Mutagenesis of cg24761295 in the *ODZ1* Promoter Reduces Gene Expression

**Figure 4:**
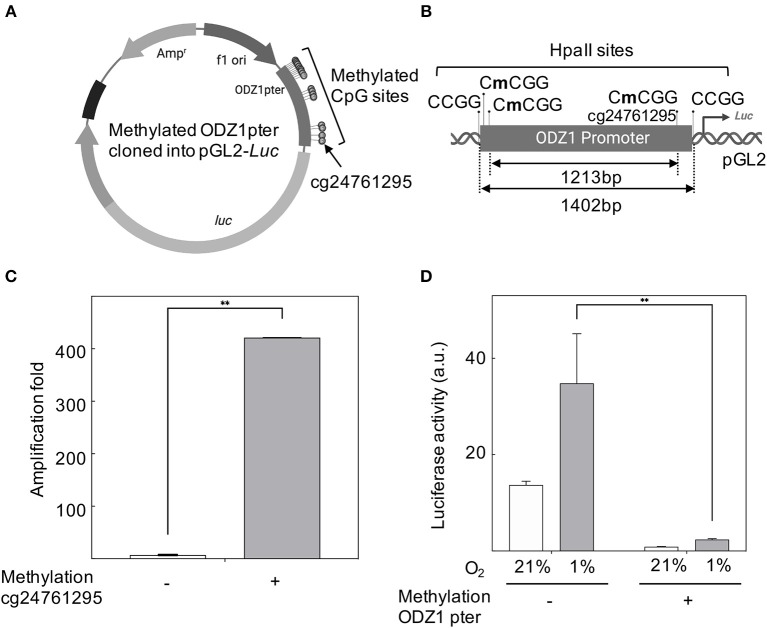
ODZ1 promoter methylation blocks gene transcription. **(A)** Schematic representation of the methylated ODZ1 promoter cloned into a pGL2-Luciferase reporter vector highlighting the methylated CpG sites, including cg24761295 (Created with BioRender.com). **(B)** Schematic diagram showing the location of HpaII sites within the ODZ1 promoter and flanking the promoter in the reporter plasmid (Created with BioRender.com). **(C)** Enzymatically methylated ODZ1 promoter was amplified with primers flanking cg24761295 after digestion with methylation sensitive HpaII. Amplification signal (uncut fragment) confirms the methylation of this site. **(D)** Luciferase activity in cells transfected with the reporter plasmid containing a methylated ODZ1 promoter and maintained under normoxia or hypoxia (48 h). Histograms show the mean ± SD of three independent experiments. Student *t*-test **p < 0.01.

**Figure 5:**
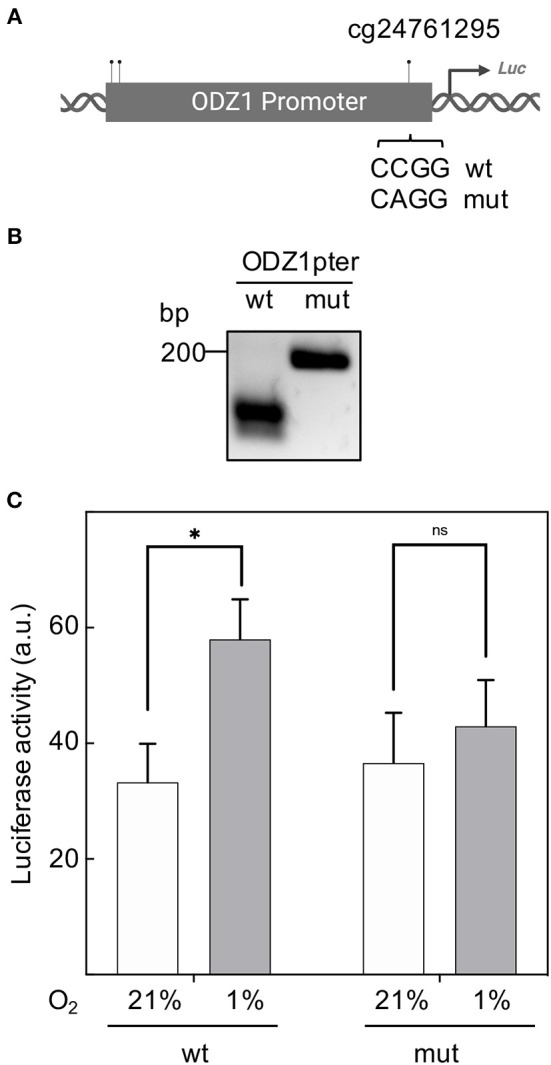
Mutation of cg24761295 reduces the transactivation capacity of the ODZ1 promoter. **(A)** Diagram shows the C to A mutation introduced in cg24761295 of the promoter (Created with BioRender.com). **(B)** A fragment of 200 bp containing cg24761295 was amplified from wild type and mutant promoter and digested with HpaII to confirm mutation. **(C)** GBM cells were transfected with wild type and mutant promoter-containing plasmids and luciferase activity was determined under hypoxia and normoxia (48 h). Histograms show the mean ± SD of three independent experiments. Student *t*-test *p < 0.05. ns, not significant.

CpG sites in the *ODZ1* promoter were enzymatically methylated by M. SssI methyltransferase prior to cloning into the luciferase reporter (Figure 4A). We confirmed the presence of methylated promoter by digesting the plasmid with HpaII, which cuts the unmethylated CCGG sites of pGL2-luciferase that flanked the promoter and leaves the methylated CCGG sites within the promoter uncut (Figure 4B). Methylation was confirmed by digesting DNA with HpaII followed by PCR amplification with primers flanking cg24761295 to assure the methylation status of this site. Only the plasmid with methylated (uncut) promoter gave an amplification signal (Figure 4C). Reporter plasmid containing an *ODZ1* methylated promoter was transfected into GBM cells and these tumor cells were cultured under hypoxia. As shown in Figure 4D, hypoxia induced luciferase activity through the unmethylated *ODZ1* promoter. However, this activity was abolished when CpG sites within the promoter were methylated. To assess the specific relevance of cg24761295, a C to A change was introduced by site-directed mutagenesis (Figure 5A) and confirmed by HpaII digestion (Figure 5B) and sequencing (not shown). Consistent with our previous results, GBM cells transfected with the wild type promoter-reporter plasmid increased luciferase activity in response to hypoxia (*p* = 0.035), whereas cells containing the mutant promoter did not (Figure 5C).

## Discussion

Cell invasion and tumor hypoxia are two key biological hallmarks associated with GBM pathogenesis and are both associated with poor prognosis (3, 19). The role of hypoxia in regulating tumor invasion through numerous molecular pathways is widely accepted (7). Nevertheless, the contribution of *ODZ1*, a gene involved in GBM invasion, to the hypoxia-induced GBM cell migration is still unknown. The tumor hypoxic microenvironment activates a number of cellular processes directed to favor an invasive GBM cell phenotype, including cytoskeletal dynamic changes that allows cell migration (7). ODZ1 plays a role in this mechanism as our group has previously described, by upregulating the RhoA/ROCK cascade that leads to cytoskeletal remodeling (14). In this study, we examined the link between ODZ1 and hypoxia in regulating tumor cell invasion and deciphered a mechanism through which hypoxia regulates ODZ1 expression and leads to tumor cell migration. In line with this, we found that ODZ1 was mainly expressed in severely hypoxic tumor regions of surgical specimens from patients with GBM. This finding was validated by *in vitro* studies demonstrating that GBM cells upregulate *ODZ1* mRNA and increased cell migration in response to hypoxia. Interestingly, blockade of ODZ1 expression by interfering RNA reduced hypoxia-induced cell migration. This finding identifies the first extracellular stimulus able to trigger the ODZ1-mediated pathway of tumor cell migration. It has been proposed that hypoxia-dependent pathways and epigenetic alterations may contribute together to the regulation of the epithelial-to-mesenchymal transition giving rise to cells with migratory capacity (20). Additionally, the hypoxic microenvironment may influence the tumor DNA methylation status. We described an increased overall methylation in GBM cells under hypoxic conditions. However, our data also indicate that CpG sites within the *ODZ1* promoter are hypomethylated under hypoxia. In support of our findings, Thienpont et al. have shown that hypoxia-induced loss of TET activity increases hypermethylation at gene promoters in GBM (21) and it has also been described that hypoxia increases the expression of a gene involved in the control of cell migration through promoter hypomethylation (22). Although the *ODZ1* promoter lacks a canonical CpG island (23–25), it does contain an aggregation of CpG sites. We have found that one of this CpG sites (cg24761295) is differentially methylated in hypoxic and normoxic GBM cells, being hypomethylated in hypoxic microenvironments. In line with this, in the TCGA database this site is commonly hypomethylated in GBM. Promoter methylation status controls gene expression through different mechanisms including histone binding or recruiting 5mC-dependent blocking proteins (26–28). Importantly, hypermethylation of *ODZ1* promoter or mutation of cg24761295 blocked the activating effect of hypoxia on ODZ1 expression, further confirming the role of this CpG site in regulating *ODZ1* promoter activity. Cumulatively, our results suggest that *ODZ1* promoter methylation has a significant role in controlling the levels of this tumor invasion-associated gene in GBM cells under hypoxia. Consistently, the 1.4 kb promoter fragment upstream of the transcription start site cloned in a reporter plasmid contained hypoxia-responsive elements, including cg24761295 at the 3' end of the promoter. Of note, cg24761295 has been shown to be hypomethylated in metastatic melanoma and colon cancer cells, compared with the primary counterparts (29) supporting the association of this CpG site with invasiveness. In line with this, we showed that methylation of CpG sites reduced the activity of the *ODZ1* promoter, being cg24761295 of major functional relevance. Besides, although overall survival has a tendential correlation with hypomethylation of cg24761295 site, this association is not statistically significant. This suggests that *ODZ1* promoter hypomethylation may not be enough to account for the *in vivo* upregulation of *ODZ1* in response to hypoxia. Other transcriptional mechanisms may also contribute to promote the expression of this cell migration-associated gene. To this end, another gene, *CDH3* (P-cadherin), also involved in cancer cell migration and invasiveness, has been shown to be upregulated by promoter hypomethylation (30) and also by transcription factors including C/EBPbeta and beta-Catenin in breast cancer cells (31, 32). Although the findings presented here support the role of hypoxia-induced epigenetic regulation of *ODZ1*, the contribution of other transcriptional pathways associated with the cellular response to hypoxia should be addressed in future studies.

The results presented here suggest that the tumor hypoxic microenvironment plays a significant role in activating the ODZ1-mediated migration of GBM cells. This activation pathway is controlled, at least in part, by the methylation status of the ODZ1 promoter being of special relevance a CpG site (cg24761295) proximal to the transcription start site. Thus, ODZ1 expression within the hypoxic tumor microenvironment may serve as a prognostic marker and therapeutic target for the clinical management of GBM patients.

## Data Availability Statement

This manuscript contains previously unpublished data. The name of the repository and accession number(s) are not available.

## Ethics Statement

The studies involving human participants were reviewed and approved by Research Ethics Board, University Health Network and Research Ethics Board, Instituto de Investigación Valdecilla. The patients/participants provided their written informed consent to participate in this study.

## Author Contributions

JF-L, GZ, and JM conceptualized, designed, and supervised the study. CV and SM managed the patient data curation. YM developed the bioinformatics methods and conducted the various analyses. SK and NT reviewed the staining and tumor specimens. OG, SM, PM, OS, and CV performed the lab work. CV, SM, and JF-L drafted the manuscript. All the authors discussed the results, co-wrote, and reviewed the manuscript.

### Conflict of Interest

The authors declare that the research was conducted in the absence of any commercial or financial relationships that could be construed as a potential conflict of interest.

## Supplementary Material

Supplementary Figure 1 **(A)** Downregulation of hypoxia-induced ODZ1 mRNA levels in G52 cells transfected with ODZ1-specific shRNAs and cultured under hypoxia for 48 h. **(B)** Cell migration capacity under hypoxia (48 h) in the presence of ODZ1 shRNAs. No significant differences in migration were obtained with the sh2 most likely due to low knock-down efficiency. Student *t*-test ^*^*p* < 0.05.

Supplementary Figure 2 Kaplan-Meier plot of the overall survival in 155 IDHwt GBM patients obtained from The Cancer Genome Atlas (TCGA) stratified according the methylation status of cg24761295 **(A)** and cg08750326 **(B)** probes in *ODZ1* promoter.

## References

[1] Stupp R Mason WP van den Bent MJ Weller M Fisher B Taphoorn MJB. Radiotherapy plus concomitant and adjuvant temozolomide for glioblastoma. N Engl J Med. (2005) 352:987–96. 10.1056/NEJMoa043330 15758009

[2] Ostrom QT Gittleman H Fulop J Liu M Blanda R Kromer C. CBTRUS statistical report: primary brain and central nervous system tumors diagnosed in the United States in 2008–2012. Neuro Oncol. (2015) 17:iv1–62. 10.1093/neuonc/nov189 26511214 PMC4623240

[3] Lefranc F Le Rhun E Kiss R Weller M. Glioblastoma quo vadis : will migration and invasiveness reemerge as therapeutic targets? Cancer Treat Rev. (2018) 68:145–54. 10.1016/j.ctrv.2018.06.017 30032756

[4] Ruiz-Ontañon P Orgaz JL Aldaz B Elosegui-Artola A Martino J Berciano MT. Cellular plasticity confers migratory and invasive advantages to a population of glioblastoma-initiating cells that infiltrate peritumoral tissue. Stem Cells. (2013) 31:1075–85. 10.1002/stem.1349 23401361

[5] Chen J Li Y Yu T-S McKay RM Burns DK Kernie SG. A restricted cell population propagates glioblastoma growth after chemotherapy. Nature. (2012) 488:522–6. 10.1038/nature11287 22854781 PMC3427400

[6] Bao S Wu Q McLendon RE Hao Y Shi Q Hjelmeland AB. Glioma stem cells promote radioresistance by preferential activation of the DNA damage response. Nature. (2006) 444:756–60. 10.1038/nature05236 17051156

[7] Monteiro A Hill R Pilkington G Madureira P. The role of hypoxia in glioblastoma invasion. Cells. (2017) 6:45. 10.3390/cells6040045 29165393 PMC5755503

[8] Chen J-WE Lumibao J Blazek A Gaskins HR Harley B. Hypoxia activates enhanced invasive potential and endogenous hyaluronic acid production by glioblastoma cells. Biomater Sci. (2018) 6:854–62. 10.1039/C7BM01195D 29485655 PMC5869158

[9] Rosa P Catacuzzeno L Sforna L Mangino G Carlomagno S Mincione G. BK channels blockage inhibits hypoxia-induced migration and chemoresistance to cisplatin in human glioblastoma cells. J Cell Physiol. (2018) 233:6866–77. 10.1002/jcp.26448 29319175 PMC13482417

[10] Wang Y Liu T Yang N Xu S Li X Wang D. Hypoxia and macrophages promote glioblastoma invasion by the CCL4-CCR5 axis. Oncol Rep. (2016) 36:3522–8. 10.3892/or.2016.5171 27748906

[11] Huang W Ding X Ye H Wang J Shao J Huang T. Hypoxia enhances the migration and invasion of human glioblastoma U87 cells through PI3K/Akt/mTOR/HIF-1α pathway. Neuroreport. (2018) 29:1. 10.1097/WNR.0000000000001156 30371540

[12] Joseph JV Conroy S Pavlov K Sontakke P Tomar T Eggens-Meijer E. Hypoxia enhances migration and invasion in glioblastoma by promoting a mesenchymal shift mediated by the HIF1α-ZEB1 axis. Cancer Lett. (2015) 359:107–16. 10.1016/j.canlet.2015.01.010 25592037

[13] Wang Z Deng M Liu Z Wu S. Hypoxia-induced miR-210 promoter demethylation enhances proliferation, autophagy and angiogenesis of schwannoma cells. Oncol Rep. (2017) 37:3010–8. 10.3892/or.2017.5511 28440459

[14] Talamillo A Grande L Ruiz-Ontañon P Velasquez C Mollinedo P Torices S. ODZ1 allows glioblastoma to sustain invasiveness through a Myc-dependent transcriptional upregulation of RhoA. Oncogene. (2017) 36:1733–44. 10.1038/onc.2016.341 27641332

[15] Aryee MJ Jaffe AE Corrada-Bravo H Ladd-Acosta C Feinberg AP Hansen KD. Minfi: a flexible and comprehensive Bioconductor package for the analysis of Infinium DNA methylation microarrays. Bioinformatics. (2014) 30:1363–9. 10.1093/bioinformatics/btu049 24478339 PMC4016708

[16] Fortin J-P Triche TJ Hansen KD. Preprocessing, normalization and integration of the Illumina HumanMethylationEPIC array with minfi. Bioinformatics. (2017) 33:558–60. 10.1101/065490 28035024 PMC5408810

[17] Mamatjan Y Agnihotri S Goldenberg A Tonge P Mansouri S Zadeh G. Molecular signatures for tumor classification: an analysis of the cancer genome atlas data. J Mol Diagn. (2017) 19:881–91. 10.1016/j.jmoldx.2017.07.008 28867603

[18] Nogueira L Ruiz-Ontañon P Vazquez-Barquero A Lafarga M Berciano MT Aldaz B. Blockade of the NFκB pathway drives differentiating glioblastoma-initiating cells into senescence both *in vitro* and *in vivo*. Oncogene. (2011) 30:3537–48. 10.1038/onc.2011.74 21423202

[19] Cavazos DA Brenner AJ. Hypoxia in astrocytic tumors and implications for therapy. Neurobiol Dis. (2016) 85:227–33. 10.1016/j.nbd.2015.06.007 26094595 PMC4684800

[20] Yeo CD Kang N Choi SY Kim BN Park CK Kim JW. The role of hypoxia on the acquisition of epithelial-mesenchymal transition and cancer stemness: a possible link to epigenetic regulation. Korean J Intern Med. (2017) 32:589–99. 10.3904/kjim.2016.302 28704917 PMC5511947

[21] Thienpont B Steinbacher J Zhao H D'Anna F Kuchnio A Ploumakis A. Tumour hypoxia causes DNA hypermethylation by reducing TET activity. Nature. (2016) 537:63–8. 10.1038/nature19081 27533040 PMC5133388

[22] Shi X Liu H Cao J Liu Q Tang G Liu W. Promoter hypomethylation of maspin inhibits migration and invasion of extravillous trophoblast cells during placentation. PLoS ONE. (2015) 10:e0135359. 10.1371/journal.pone.0135359 26263377 PMC4532475

[23] Esteller M. CpG island hypermethylation and tumor suppressor genes: a booming present, a brighter future. Oncogene. (2002) 21:5427–40. 10.1038/sj.onc.1205600 12154405

[24] Unruh D Zewde M Buss A Drumm MR Tran AN Scholtens DM. Methylation and transcription patterns are distinct in IDH mutant gliomas compared to other IDH mutant cancers. Sci Rep. (2019) 9:8946. 10.1038/s41598-019-45346-1 31222125 PMC6586617

[25] Ando M Saito Y Xu G Bui NQ Medetgul-Ernar K Pu M Chromatin dysregulation and DNA methylation at transcription start sites associated with transcriptional repression in cancers. Nat Commun. (2019) 10:2188 10.1038/s41467-019-10557-7 31097695 PMC6522544

[26] Baylin SB Esteller M Rountree MR Bachman KE Schuebel K Herman JG. Aberrant patterns of DNA methylation, chromatin formation and gene expression in cancer. Hum Mol Genet. (2001) 10:687–92. 10.1093/hmg/10.7.687 11257100

[27] Baylin SB. DNA methylation and gene silencing in cancer. Nat Clin Pract Oncol. (2005) 2:S4–11. 10.1038/ncponc0354 16341240

[28] Herman JG Baylin SB. Gene silencing in cancer in association with promoter hypermethylation. N Engl J Med. (2003) 349:2042–54. 10.1056/NEJMra023075 14627790

[29] Vizoso M Ferreira HJ Lopez-Serra P Carmona FJ Martínez-Cardús A Girotti MR. Epigenetic activation of a cryptic TBC1D16 transcript enhances melanoma progression by targeting EGFR. Nat Med. (2015) 21:741–50. 10.1038/nm.3863 26030178 PMC4968631

[30] Paredes J Albergaria A Oliveira JT Jerónimo C Milanezi F Schmitt FC. P-cadherin overexpression is an indicator of clinical outcome in invasive breast carcinomas and is associated with CDH3 promoter hypomethylation. Clin Cancer Res. (2005) 11:5869–77. 10.1158/1078-0432.CCR-05-0059 16115928

[31] Albergaria A Resende C Nobre AR Ribeiro AS Sousa B Machado JC. CCAAT/enhancer binding protein β (C/EBPβ) isoforms as transcriptional regulators of the pro-invasive CDH3/P-cadherin gene in human breast cancer cells. PLoS ONE. (2013) 8:e55749. 10.1371/journal.pone.0055749 23405208 PMC3566012

[32] Faraldo MM Teulière J Deugnier M-A Birchmeier W Huelsken J Thiery JP. beta-Catenin regulates P-cadherin expression in mammary basal epithelial cells. FEBS Lett. (2007) 581:831–6. 10.1016/j.febslet.2007.01.053 17292359

