# Supplementary figures and images for: Hypoxia Can Induce Migration of Glioblastoma Cells Through a Methylation-Dependent Control of ODZ1 Gene Expression
Source: Front Oncol. 2019 Oct 10;9:1036. doi: 10.3389/fonc.2019.01036 (PMC6795711; doi:10.3389/fonc.2019.01036)

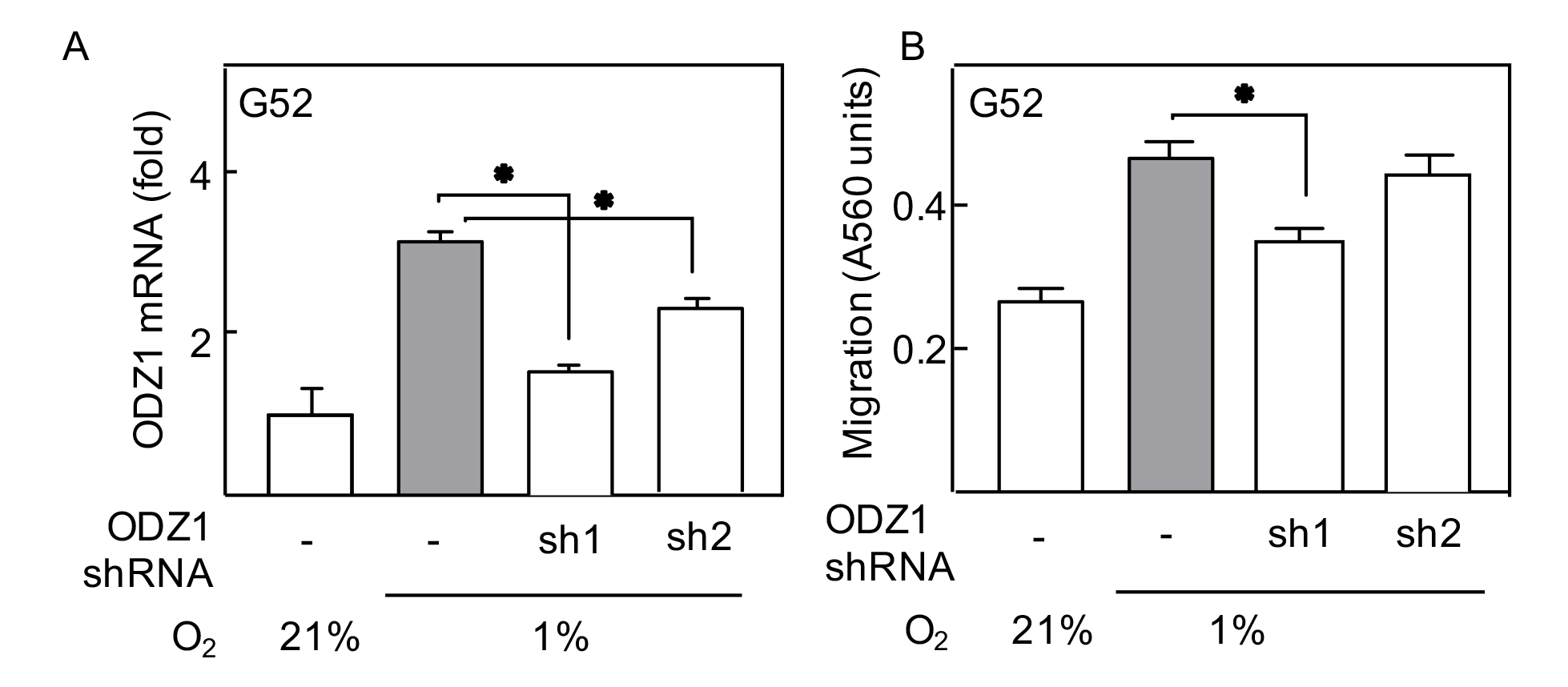

Supplement: Supplementary Figure 1 — (A) Downregulation of hypoxia-induced ODZ1 mRNA levels in G52 cells transfected with ODZ1-specific shRNAs and cultured under hypoxia for 48 h. (B) Cell migration capacity under hypoxia (48 h) in the presence of ODZ1 shRNAs. No significant differences in migration were obtained with the sh2 most likely due to low knock-down efficiency. Student t-test *p < 0.05. [file Image_1.TIF]

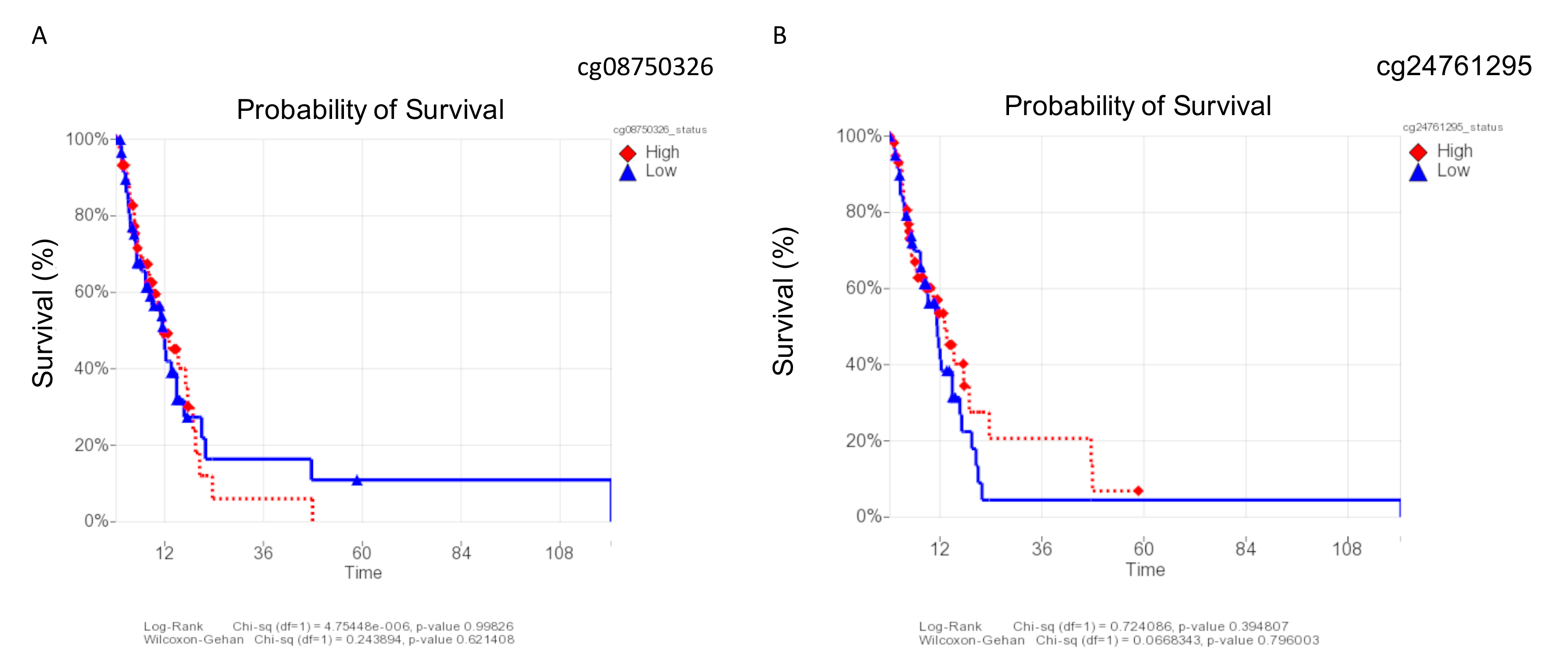

Supplement: Supplementary Figure 2 — Kaplan-Meier plot of the overall survival in 155 IDHwt GBM patients obtained from The Cancer Genome Atlas (TCGA) stratified according the methylation status of cg24761295 (A) and cg08750326 (B) probes in ODZ1 promoter. [file Image_2.TIF]
